# Correction

**DOI:** 10.1093/ofid/ofad489

**Published:** 2023-10-06

In the below-listed manuscripts, a change has been made to the author list.

- https://doi.org/10.1093/ofid/ofz061
- https://doi.org/10.1093/ofid/ofy182
- https://doi.org/10.1093/ofid/ofy289

